# A Serious Game for the Prevention of Obesity in School Children–Impact of Parent’s Involvement: A Randomized Controlled Trial

**DOI:** 10.3390/life12060779

**Published:** 2022-05-24

**Authors:** Alisa Weiland, Nadine Reiband, Norbert Schäffeler, Guido Zurstiege, Katrin Elisabeth Giel, Stephan Zipfel, Isabelle Mack

**Affiliations:** 1 Department of Psychosomatic Medicine and Psychotherapy, University Medical Hospital, 72076 Tübingen, Germany; nadine.reiband@gmx.de (N.R.); norbert.schaeffeler@med.uni-tuebingen.de (N.S.); katrin.giel@med.uni-tuebingen.de (K.E.G.); stephan.zipfel@med.uni-tuebingen.de (S.Z.); 2 Department of School Psychology, University of Tübingen, 72076 Tübingen, Germany; 3 Department of Media Studies Tübingen, University of Tübingen, 72076 Tübingen, Germany; guido.zurstiege@uni-tuebingen.de

**Keywords:** children, serious game, nutrition, family involvement, psychology

## Abstract

Serious games convey information and use interactive components to reinforce and train behaviours. A serious game addressing nutrition, physical activity and stress coping—the Kids Obesity Prevention Program (KOP)—was previously evaluated for efficacy in children. This study aimed at evaluating the KOP-game regarding: (i) its acceptance and efficacy with respect to parents of primary school children receiving the same game intervention as the children; and (ii) whether the children could benefit by parental involvement. A randomized controlled trial with two groups of children aged 9 to 12 years was conducted which included a 6-month follow-up period. All children played the game twice in two weeks. In the family-intervention group, the parents additionally played the game. The primary outcome was the gain in knowledge in parents and children measured with a pretested questionnaire. The secondary outcomes were knowledge maintenance as well as several behavior changes. Parents and children in both groups improved and maintained their knowledge equally. The KOP-game increases knowledge of nutrition in children independently of the involvement of their parents. KOP games are well accepted in children; further research should examine the structured involvement of parents.

## 1. Introduction

Obesity is a severe public health problem for children and adults [1]. Many school-aged children are already overweight or obese [2,3] and most of them will remain overweight and obese in adulthood [4]. Therefore, it is important to establish prevention and treatment strategies for effective weight-management in childhood.

Existing weight-management programs for the treatment of childhood obesity focus on improving diet and eating behaviour, on the one hand (e.g., by decreasing portion sizes and/or the energy density of foods and beverages), and increasing physical activity, on the other. Moreover, it is well known that psychological and psychosocial aspects play a critical role in behavioural changes, determining the success of intervention programs. In fact, family support is extremely important for changes in children’s behavior: one important basic requirement of weight-management programs is that both children and their families are motivated and show goal commitment with respect to a behaviour change [5,6].

Approaching children within their familiar environment by using new media tools could therefore be an important component to lower the barriers to such programs. The technical facilities are available in most families; their use is well-known and they belong to the world of children’s everyday life. The extent of the development in e-health in recent decades has been enormous. There are many well-accepted games that facilitate health-education by: (1) improving knowledge about nutrition, eating habits and exercise (serious games); (2) increasing physical activity (exergames); or (3) combining both approaches [7]. Regarding diet, balancing portion sizes and dietary energy density (DED) are the two key factors. However, patients with obesity have increased gastric capacities; thus, larger portions are needed to induce satiety in comparison to normal-weight controls [8]. Moreover, a recent meta-analysis showed a significant correlation between body weight reduction and the dietary energy density (DED) of food intake in people with obesity [9]. Additionally, using the food pyramid or the MyPlate concept to guide portion sizes is associated with limitations regarding personal preferences. Thus, the DED appears to be an important factor for dietary adjustments in obesity. The DED principle is already understood by children aged 9–11 years old [10]. To date, there has been only one serious game evaluated that explicitly teaches the DED principle—the Kids Obesity Prevention Program (KOP) [10,11]—although the topic is also indirectly the focus of other games [11,12,13].

A systematic review of games targeting obesity prevention and treatment [11] showed that, at a qualitative level, most studies with serious games reported positive effects on obesity-related outcomes (improvement of weight-related parameters, physical activity or dietary behavior/knowledge), but the observed effects were small. For serious games, follow-up investigations were rarely reported and maintenance of knowledge after playing games promoting the DED principle has never been tested over a long time period [11].

Interestingly, only one third of the studies published up to 2017 included parents, mostly only for data collection, despite child–parent interactions being very important, as parents are role models for their children. Since 2017, the situation has not improved [7]. If studies included parents, these were mostly exergames. Regarding serious games, the parents sometimes received additional newsletters or websites but not the children’s intervention, so that contentual exchange was rather difficult [11]. Interestingly, Baranowski and colleagues (2012) showed that it is very important to give clear instructions for parental involvement and that it is not sufficient to only provide the materials [14].

KOP is a motion-controlled serious game and was developed as an additional educational component for the prevention and treatment of childhood obesity. It addresses all the three core areas: nutrition, physical activity and stress coping [10]. In addition to motion control as a component of physical activity, a tablet is used for knowledge-based and cognitive tasks. In comparison with other games, the nutrition aspect not only deals with the food pyramid but also with the energy density of foods and liquids. Moreover, psychological aspects, especially stress and stress-coping strategies, are addressed. A cluster-randomized study for the evaluation of the efficacy of KOP showed that children between 9 and 12 years of age were able to understand and to utilize this concept and to improve their nutritional knowledge significantly compared with a control group. More importantly, the knowledge was sustained over a period of 4 weeks, which could be explained by the high level of challenging interactions, the number of repetitions and self-reflection tools applied in the game. However, parents in this study were only involved in data assessment.

### Objective and Hypothesis

The aim of this KOP-2 study was to evaluate the KOP-game regarding: (i) its acceptance and efficacy in parents of primary school children receiving the same game intervention as the children; and (ii) whether the children could benefit by the involvement of their parents. Therefore, a randomized controlled trial with two parallel groups in a primary school involving children aged 9 to 12 years was conducted which included a 6-month follow-up period. The primary outcome was the gain in knowledge about important lifestyle factors, with a focus on the DED principle in the nutrition section. Secondary outcomes were knowledge maintenance and changes in behaviours (eating behaviour, physical activity, media consumption) and acceptance of the game.

The primary hypothesis was that at the termination of the intervention, parents in the family-intervention group (FI-group; children and parents received game intervention) would have higher knowledge scores compared to the child-intervention group (CI-group; only children received game intervention). Further, we hypothesized that all children would improve their knowledge from baseline to the end of the intervention and that this knowledge improvement would be maintained long-term. Furthermore, the intervention would be well accepted. Due to minimal intervention, no behaviour changes were assumed regarding media consumption, eating behaviour or physical activity in the groups independently of the intervention.

## 2. Materials and Methods

### 2.1. Study Design and Participants

A prospective randomized controlled trial with 46 4th-grade school children aged 9 to 11 years and their parents was conducted from November 2016 to July 2017 in Germany. Recruitment of children took place at a parent’s evening for the school classes. Parents as well as children provided written informed consent.

The participants were randomly grouped into a family-intervention group (FI) (*n* = 23) and a control group with only child-intervention (CI) (*n* = 23) using Randlist, DatInf GmbH, Tübingen, Germany, before the commencement of the study. Owing to the nature of the intervention, participants and outcome assessors could not be blinded to treatment assignments. Two children of the CI-group became ill before the intervention started, so the final group size was *n* = 21. The study period was 2 weeks and follow-ups were conducted 4 weeks and 6 months after study termination. The inclusion criterion was an age between 9 and 12 years (all children who were in the 4th grade of primary school). Major linguistic difficulties in children or parents were an exclusion criterion. Due to ethical reasons, exclusion was conducted after study participation.

The study protocol was approved by the Ethics Committee of the medical faculty of the Eberhard Karls University Tübingen and the University Hospital Tübingen, Germany (050/2014BO1). The KOP-2 study is registered at Clinicaltrials.gov (NCT02942823).

### 2.2. Treatments 

*Family-Intervention Group (FI-group)*: The children played the game twice at school in a two-week period. The duration of each session was 45 min. An investigator was present the whole time. The intervention consisted of the KOP-1 [10] game but without the motion-control. Additionally, the games were taken home on a tablet computer and the parents received instructions to play the games according to their liking, either alone or together with their children or partner. They had the possibility to stop gaming and proceed later but were instructed to play the whole game at least once, which takes between 60 and 90 min. 

The topics addressed by the game belong to the categories of nutrition, physical activity and stress coping. The nutrition segment of the game deals with the food pyramid and the sugar contents of liquids and focuses on factors which are important for satiety, therefore teaching the concept of DED-P extensively [10]. In short, the game consists of the following mini-games: (I) Pack Your Backpack with Food: The participants are asked to pack as many foods into their virtual backpack as they may need in a day (breakfast, lunch, dinner and two mid-meals). Afterwards, they receive individualized feedback, considering their individual energy expenditure and the distribution of the selected foods according to food groups; (II) Balloon Game: The participants are asked to sort several foods into their food groups by popping flying balloons at the right moment; (III) Food Under the Microscope: In the laboratory, the participants learn to group foods into three categories (green: energy density smaller than 150 kcal/100 g; red: energy density greater than 250 kcal/100 g; yellow: in-between energy density). They learn about the composition of foods regarding water, fat, carbohydrates, protein and fiber. After this learning session, they have to organize the foods by themselves into the three categories according to their energy density; (IV) Liquid Rankings on the Sugar Scale: In this game, several liquids are presented and the participants are asked to guess how many lumps of sugar each liquid contains; (V) Kangaroo–Turtle Race: In this game, participants are asked to help a turtle to win a race against a kangaroo by choosing the food with a lower energy density out of known and unknown food pairs under time pressure. With a correct decision, the rocket on the turtle is activated; if the answer is false, there is a penalty of three seconds. 

A detailed overview of all game modules is provided in KOP-1 [10].

*Child-Intervention Group (CI-group)*: In the CI-group, the children received the same intervention as the children in the FI-group. However, the parents were not involved.

### 2.3. Outcome Measures

At baseline (T0), one day after study completion (T1) and at 4 weeks (T2) and 6 months (T3) follow-up, measurements were performed in children and their parents. 

The primary outcome of the study was the gain in knowledge (nutrition, stress) in parents and children, analyzed between the CI-group and the FI-group at T0 versus T1. Knowledge about a healthy lifestyle, with a focus on nutritional issues, especially the DED principle, was tested using the pretested questionnaire tailored specifically to the serious game [10]. For adults, the questionnaire had to be slightly modified for practical reasons. The knowledge questionnaire was transformed into scales ranging from 0 to 60 for children and 0 to 45 for parents. The total knowledge test score included all items; the dietary energy density score included items 3, 5, 7 and 9; the food pyramid score included the items 1, 2 and 4; and the stress score included the items 10, 11, 12 and 13. The original questionnaire is provided in Supplementary File S1.

Secondary outcomes were the maintenance of knowledge in children and their parents at T2 and T3, the acceptance of the game and changes in dietary behaviour and physical activity, measured by mostly validated questionnaires.

An extensive overview of the applied measurements for the secondary outcomes is provided in KOP-1 [10]. Briefly, the instruments, along with minor changes in relation to the KOP-1 study, are reported below.

*Maintenance of knowledge* was measured by applying the knowledge questionnaire as described above at the 4-week and 6-week follow-up after T2 (T3) for children and adults.

*Acceptance of the game (children and parents of FI at T1)* was measured by the following item: “Overall, I like the game”, answered on a 4-point scale.

*Changes in dietary behavior* [15] *(CI and FI at T0, T2 and T3)* were measured using the an index for healthy nutrition [16]. This index consists of food items which are considered to be indicators for healthy and unhealthy eating behaviours. The indicator food items used for this index are: vegetables and fruits (cooked, raw, frozen, tinned), whole-grain bread, soft drinks, fast food, chocolate and snacks, such as crisps or pretzel sticks. The questions were taken from the corresponding validated food frequency questionnaire [15]. The children completed the questionnaire by themselves, with the parents also completing the same questionnaire for their children.

*Physical activity (CI and FI at T0, T2 and T3)* was measured by a validated questionnaire [17] which was completed by the children themselves and by the parents on behalf of their children. The questionnaire consisted of seven items, each with six answer options. A score was calculated which allowed the categorization of activity levels into low, medium and high.

*Media consumption (CI and FI at T0, T2 and T3)* was measured by questions from the KiGGS Questionnaire [18]. Four questions ask about the average time the child spends watching television or playing video games, or about time spent on the computer during the week and at weekends. The questions each have five response options. 

*Procedure of measurements:*

Data were collected using standard operating procedures. All questionnaires were completed by all participating children simultaneously in a classroom setting with separated tables using paper and pencil. A teacher and a trained school psychologist were present at all times during this process. No specific instructions were given to individual children. The parents completed the questionnaires at home using paper and pencil. The BMI z-score was calculated by calculating the BMI on the basis of body weight measured by a calibrated scale and body height measured by a stadiometer and with reference to age- and sex-specific norms [19].

### 2.4. Sample Size

The sample size was calculated with regard to the primary outcome, the group (FI vs. CI)-by-time (T0 vs. T1) interaction of knowledge in parents. Estimating conservatively an effect size of 0.25, we aimed to recruit 17 parents in each group to reach a power of 80% at the alpha level of 0.05, as calculated with G-Power [20]. We assumed a drop-out rate of roughly 15% due to illness or for other reasons in the school setting. Additionally, we expected that in most families both parents would not take part in the study. Thus, we decided to include 20 children and their parents (ideally, 40) in each group, resulting in 40 children and, ideally, 80 parents, overall. 

### 2.5. Statistical Analysis 

Data were analyzed using SPSS version 25. Data are presented as means (standard deviation) and frequencies are given as percentages (%) unless stated otherwise. Prior to test statistics, the normality distribution of the data was tested using the Kolmogorov–Smirnov test, along with the equality of variances between groups, using Levene’s test. Baseline differences between the groups were analyzed using unpaired *t*-tests—by the Mann–Whitney U test if non-parametric, or, if non-metric, by the χ^2^ test or the Fisher–Freeman–Halton test [21].

*Primary Outcome:* Calculation of group (FI vs. CI) × time (T0 vs. T1 for knowledge in children and adults) interaction by 2 × 2 ANOVA. 

*Secondary Outcomes:* Calculation of group (FI vs. CI) × time (T0 vs. T1 vs. T3 for knowledge and other variables) interaction by 2 × 3 ANOVA. 

*Handling of missing data for single items in questionnaires at baseline (secondary outcomes)*: At baseline, all parents and children filled in questionnaires as secondary outcome variables, but there were instances of single questions of questionnaires not being answered. The predictive mean matching method (PMM) [22,23,24], with 5 cases in each match set, was used for missing data (mis-d) imputation for the knowledge score, parent version (mis-d: 28.26) and child version (mid-d: 4.2%); the food frequency questionnaire (FFQ), parent version (mis-d: 4.5%) and child version (mis-d: 8.2%); the activity questionnaire, parent version (mis-d: 4.7%) and child version (mis-d: 5.3%); and questions about media consumption (mis-d: 15.7%).

*Intention to treat (ITT) analysis and handling of missing data for single items in questionnaires at the end of the study period and follow-up*: We analyzed the primary and secondary outcomes by intention to treat (ITT) analysis using the PMM, with 5 cases in each match set. Mis-d for single items in the questionnaires were imputed as described for the handling of missing data at baseline. The percentages of mis-d for the questionnaires at T1 were as follows: Knowledge Score: parent version, 35.2%; child version, 2.2%. At T2, they were: Knowledge Score: parent version, 55.2%; child version, 10.1%; FFQ: parent version, 32.1%; child version, 15.7%; activity questionnaire: parent version, 33%; child version, 11.2%; and questions about media consumption: parent version, 32%; child version, 13.9%. At T3, they were: Knowledge Score: child version, 28.0%; FFQ: parent version, 43.1%; child version, 30.1%; activity questionnaire: parent version, 44.1%; child version, 28.6%; and questions about media consumption: 33%.

## 3. Results

### 3.1. Participant Characteristics

**Figure 1:**
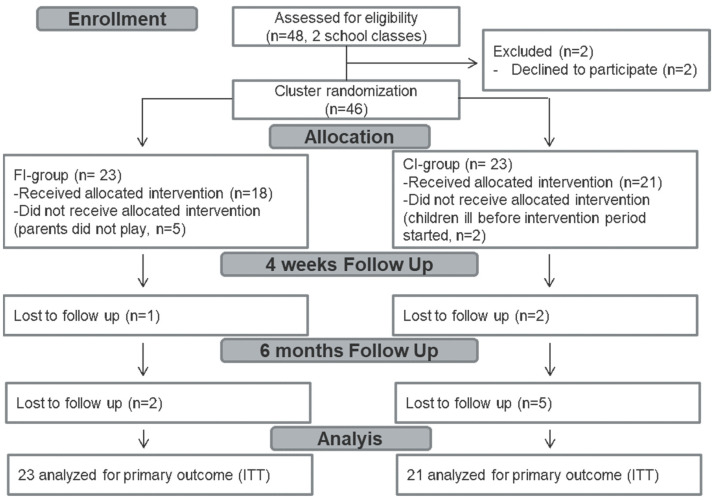
Consolidated Standards of Reporting Trials 2010 flow diagram. ITT: intention to treat, FI: family intervention, CI: child intervention.

The trial flow of the participants is shown in Figure 1. In the FI-group, all 23 children played the game at school, but 5 out of the 23 families (22% drop-out) did not play the game at home. In the CI-group, 21 out of the 23 allocated children played the game at school and the two children who did not play dropped out before the intervention period started due to illness. Table 1 shows the baseline characteristics of the 21 children in the CI-group and the 23 children in the FI-group included in the ITT analysis. Additionally, the baseline characteristics of the parents are shown. The children in the groups did not differ with respect to age, sex, BMI *z*-score, knowledge score, self-reported eating behavior, media consumption, intentions to live healthily or self- or parent-reported physical activity. However, children in the FI-group showed significantly less favorable parent-reported eating behavior than children in the CI-group. There were no differences at baseline in parent characteristics between the two groups (Table 1).

### 3.2. Outcomes

The data for the primary and secondary outcomes with intention to treat analysis are presented in Table 2 and Table 3.

#### 3.2.1. Primary Outcome

*Knowledge gain in parents and children* (total knowledge test score) was analyzed as per protocol. Parents in the FI-group received additional intervention, while parents in the CI-group received no intervention. There was a significant increase in parents’ total knowledge scores from T0 (FI-group: 62%; CI-group: 60%) to T1 (FI-group: 67%; CI-group: 68%), F(1,40) = 24.61, *p* < 0.001, independently of group allocation, F(1,40) = 0.27, *p* = 0.607.

Also analyzed with intention to treat, knowledge (total knowledge test score) emerged from T0 (FI-group: 62%; CI-group: 60%) to T1 (FI-group: 66%; CI-group: 69%), F(1,76) = 16.31, *p* < 0.001, independently of group allocation, F(1,76) = 1.82, *p* = 0.182. 

For children, the intervention was similar in the FI-group and the CI-group; both received the health game intervention. Analyzed as per protocol, there was a significant increase in children’s total knowledge scores from T0 (FI-group: 46%; CI-group: 46%) to T1 (FI-group: 66%; CI-group: 62%), F(1,36) = 77.21, *p* < 0.001, independently of group allocation, F(1,36) = 18.56, *p* =0.414.

Analyzed with intention to treat, the children in the FI-group improved their averagely knowledge significantly from baseline (27.24; 45%) to T1 (38.00, 63%), as did those in the CI-group (baseline: 27.67, 46%; T1: 37.00, 62%), F(1,40) = 47.52, *p* < 0.001. This knowledge gain was independent of group allocation, F(1,40) = 1, *p* = 0.398. 

#### 3.2.2. Secondary Outcomes 

*Maintenance of knowledge in parents* was tested at the 4-week follow-up from T1 (T2). The gain in knowledge was maintained in the FI-group (30.07, 67%) and the CI-group (29.91, 66%) over a 4-week period independently of group allocation.

*Maintenance of knowledge in children* tested at the 4-week follow-up from T1 (T2) and at the 6-month follow-up (T3) was maintained in the FI-group over a 4-week period (37.53, 63%) and over the 6-month period (37.35, 62%). The CI-group maintained the knowledge over the 4 weeks (35.48, 59%) and over the six months (34.71, 58%) as well. 

*Acceptance of the game* was assessed after the game session (T1). The acceptance of the game by all children was high: 78% of the CI-group reported that they liked the game, 10% were uncertain and only 5% disliked the game. Similar results were found in the FI-group, in which 87% liked the game and none disliked it. Out of the parents in the FI-group, 40% liked the game and 44% disliked the game.

*Dietary behavior* was assessed using a healthy nutrition index (HNI). At baseline, the HNI was predominantly neutral or favorable as reported by the parents (0% unfavorable in both groups). Following the children’s report, there were significant baseline differences in the HNI: 36% in the FI-group and 7% in the CI-group had and unfavorable HNI at baseline. This group difference disappeared after intervention at the follow-up at T2 (FI-group: 4%; CI-group: 0%) and T3 (FI-group: 0%; CI-group: 9%). However, the change in the HNI over time was not significant independently of the source. 

*Physical activity:* The information from children and parents differed enormously at baseline: Children in the FI-group reported themselves as being mostly low active (52%), whereas the parents reported them as mostly medium active (65%); low activity was only reported in 13%. In the CI-group, most of the children had a moderate (59%) physical activity level reported by parents and a high amount of low activity (52%) reported by themselves. So, in both groups the parents reported higher levels of children’s activity than the children themselves. No changes were observed in the time course between the different group allocations as reported by children and their parents (Table 4 and Table 5). 

*Media consumption:* Screentime on weekdays was between 0 and 30 min or 1–2 h for all children in the FI-group and 88% of the children in the CI-group at baseline. The amount of TV-watching emerged in the FI-group as 1–2 h per day for 11% (T2) and 5% (T3) of the children; this time effect was not significant. Gaming activities at a computer on weekdays was between 0 and 30 min for all children at nearly all timepoints. No significant changes were found between the groups in the time course. Detailed data are reported in Table 2 and Table 3.

## 4. Discussion

This randomized controlled trial aimed at evaluating the previously evaluated KOP-1 [10] game regarding: (i) its acceptance and efficacy in parents and their children; and (ii) whether the children could benefit by the involvement of their parents.

The primary outcome was the gain in knowledge in the thematic fields addressed by the serious game in parents and their children. Against our hypothesis, the gain in knowledge in the FI-group was not superior to the CI-group. Parents in both groups slightly improved their knowledge independently of the intervention from baseline. One possible explanation could be an iteration effect combined [25] with a ceiling effect. Parents achieved on average 60% of the knowledge score at baseline, which is already high when compared with the children (45%), the latter achieving on average around 60% after the completed intervention. The overall time effect in favor of parental knowledge in both groups may be due to more awareness with regard to the addressed game topics simply by completing the questionnaires due to an iteration effect [25] or due to interactions with their children [26], the latter having received the same intervention at school in both trial arms.

Additionally, children improved their knowledge about nutrition independently of group allocation. This was, on the one hand, assumable because all children received the game-play intervention. On the other hand, interactions between parents and children in the FI-group could have been expected to deepen and enhance the knowledge of children. However, only small effects would have been likely, and the sample size is too small to detect these. Additionally, it needs to be considered that the degree of involvement at qualitative and quantitative levels by the parents could not be completely controlled since the interactions took place at home. In detail, neither the amount of game playing together with their children nor talking to them about the game play and exchanging experiences could be controlled, whereas the game play of children was conducted under controlled conditions at school. In comparison with other studies, we performed a 4-week and 6-month follow-up study [11]. We found that the amount of knowledge remained high in children and parents, suggesting that at the cognitive level, knowledge, especially about nutrition and the DED principle, was sustainable, as previously shown in KOP1 [10], though such findings are rather rare in the existing literature. Knowledge is quickly lost if not consciously reviewed from time to time [27,28]. The high number of challenging interactions and repetitions due to the applied games among children and parents in the FI-group and repeated questionnaires among all participants could be a possible explanation.

At the behavioral level, the groups were similar at baseline, except for the healthy nutrition index reported by children. This index was more unfavorable in the FI-group than in the CI-group. These differences disappeared after intervention at T1. Thus, the intervention may have positively influenced the children’s dietary behaviour. However, this result should be interpreted with caution, since the healthy nutrition index reported by parents on behalf of their children was always similar between groups. For the latter, we need to consider that parents do not completely control the food intake of their children at this age due to the increase in food intake outside of the home. Overall, the intervention time was short and large changes regarding behaviour change were unlikely to have occurred [10,29], as was borne out by our findings.

As reported previously [10], acceptance of the game was high in children. In line with the findings of Mack et al. (2017) [11], children like to learn with serious games [12,30]. In parents, acceptance was lower than in children. Of course, this may be simply due to the fact that the game was designed for children. This would be in line with the data showing slight but not impressive increases in the gain in knowledge from baseline similar to the control group. The game may not have been challenging enough for adults. Furthermore, it could be also that the game play was perceived as an additional burden, at least for some parents, added to duties of managing work and families at the same time. This may be reflected in the drop-out rate of 22% in the FI-intervention group, due to parents not playing the game at all, and missing data due to parents refusing to fill out the knowledge and other questionnaires twice, before and after intervention. However, there are many studies that show that serious games are useful in adult interventions regarding health issues. E-health games have been used to improve levels of physical activity and nutritional knowledge in adults, too [31,32]. Additionally, there are several studies that have investigated parental involvement in childhood and adolescent overweight and obesity e-health interventions [33].

This study has several strengths besides limitations. A clear strength is the study design, conducted as a cluster randomized controlled trial with 4-week and 6-month follow-ups and the inclusion of parents. As outlined in the introduction, game interventions conducted among children that include parents are scarce. The limitations are that game time at home (except from minimum game play) and interactions of parents with children and other family members were not controlled, along with the nature of the study not allowing for double-blinding and a placebo control. These factors may have influenced the results. Although parents were clearly instructed, as recommended by Baranowski et al. [14], the compliance of parents was only moderate considering the drop-out rate and completion of questionnaires. Finally, it is well known that knowledge improvements regarding health issues do not necessarily align with behavioral changes. To improve life-style changes, multi-component interventions are necessary and e-health may be one of these components. In the field of serious games, modern game designs allowing interactions between families may be promising and need further investigation and evaluation [33]. Serious games can be applied to convey health-related information and can also use interactive components to reinforce information and train behaviours, for example, by incorporating cognitive bias therapy into games. Future studies in this field involving families warrant well-considered game and study designs. Performing qualitative research in order to analyze the needs and wishes of families with respect to such game interventions should be strongly considered ahead of fully powered trials.

## 5. Conclusions

Involving parents in interventions addressing life-style factors in young school children has been considered to be an important factor for success. Therefore, this study tested the acceptance and efficacy of a previously evaluated serious game for children addressing the topics of nutrition, physical activity and stress coping in parents and whether or not children benefited by the involvement of their parents. The primary outcome, that parental knowledge would be improved after playing the KOP game, was negative. The baseline knowledge of parents was already high and children in the FI-group did not further profit from the game play of their parents in terms of their own knowledge scores. However, the KOP-game lead to sustained knowledge gain in children up to 6 months in the addressed fields, in the dietary section focusing on the DED principle. The children highly accepted the game. Additionally, the more unfavorable healthy eating index among children in the FI-group at baseline aligned with values for the CI-group after intervention. Overall, the game is suitable, especially for children, as an additional component in prevention and possibly also treatment. Involving parents appears not to provide further beneficial effects in the examined context.

## Figures and Tables

**Table 1:** Baseline characteristics of the study population.

Children’s Characteristics	FI-Group (*n* = 23)	CI-Group (*n* = 21)	*p*-Value
Age (years), median, M (SD), (range)	9.5, 9.67 (0.6) (9–11)	9.5, 9.67 (0.33) (9–10)	0.842
Sex m; f n (%)	9 (39.1); 14 (60.9)	9 (42.9); 12 (57.1)	0.802
Weight (kg), M (SD), (range)	37.05 (9.32), (21–67)	36.05 (6.6), (27–56)	0.687
Height (cm), M (SD), (range)	143.73 (9.03) (128–164)	143.89 (5.27) (128–164)	0.834
BMI *z*-score, M (SD), (range)	17.84 (4), (12.82–32.76)	17.32 (2.98), (13.50–26.27)	0.648
Normal Weight n (%)	15 (68%)	15 (79%)	-
Thinness n (%)	3 (14%)	2 (11%)	
Overweight n (%)	3 (14%)	1 (5%)	
Obesity n (%)	1 (4%)	1 (5%)	
Knowledge score M (SD) %			
Total score (max 60)	26.68 (5.08) 45%	27 (7.02) 45%	0.944
Food pyramid score (max 26)	13.07 (2.85) 50%	13.81 (3.25) 53%	0.902
Energy density score (max 15)	6.39 (2.68) 43%	5.71 (3.18) 38%	0.448
Stress score (max 15)	6.11 (2.35) 41%	6.69 (1.69) 45%	0.759
**HNI parent report M (SD)**	**9 (1.60)**	**10.18 (1.60)**	0.029 *
Unfavorable n (%)	0 (0%)	0 (0%)	
Neutral n (%)	16 (76%)	10 (59%)	
Favorable n (%)	5 (23%)	7 (41%)	
HNI Child report M (SD)	9.45 (1.75)	10.14 (2.32)	0.422
Unfavorable n (%)	0 (0)	1 (7)	
Neutral n (%)	8 (73)	6 (43)	
Favorable n (%)	3(27)	7 (50)	
Physical activity parent report			
Score M (SD)	−0.33 (2.03)	0.06 (2.36)	0.585
Low n (%)	3 (14)	3 (18)	
Medium n (%)	15 (72)	10 (59)	
High n (%)	3 (14)	4 (23)	
Physical activity child report			
Score M (SD)	−1.11 (2.87)	−0.53 (2.70)	0.54
Low n (%)	12 (57)	11 (52)	
Medium n (%)	6 (29)	6 (29)	
High n (%)	3 (14)	4 (19)	
Screentime watching n (%)			0.141
≤0.5 h	10 (48)	11(58)	
1–2 h	11 (52)	6 (32)	
≥3 h	0 (0)	2 (10)	
Screentime gaming n (%)			0.631
≤0.5 h	19 (95)	15 (83)	
1–2 h	1(5)	3 (17)	
≥3 h	0 (0)	0 (0)	
Intentions to live healthy			
Score (max 65) M (SD)	26.65 (7.06)	25.63 (6.45)	0.684
**Parents’** **Characteristics**	**FI-Group (*n* = 30)**	**CI-Group (*n* = 33)**	***p*** **-Value**
Age (years), Median, M (SD), (range)	42, 42.01 (3.68) (36–47)	42, 42.3 (5.12) (36–52)	0.876
Sex m; f n (%)	12 (38.7); 18 (61.3)	14 (42.4); 19 (57.6)	0.659
Weight (kg), Median, M (SD), (range)	77, 78.2 (8.1), (63–88)	79, 77.2 (6.54), (68–89)	0.687
Height (cm), M (SD), (range)	173.2 (6.54) (161–184)	173.5 (3.72) (167–180)	0.897
BMI *z*-score, M (SD), range	26.1 (6.54) (22.8–32)	25.6 (2.47) (22.3–30.6)	0.775
Normal weight n (%)	18 (60)	21 (64)	-
Thinness n (%)	5 (17)	2 (6)	
Overweight n (%)	4 (13)	8 (24)	
Obesity n (%)	3 (10)	2 (6)	
Knowledge score (both) M (SD)			
Total score (max 45)	27.57 (3.60) 61%	26.93 (5.80) 60%	0.544
Food pyramid score (max 11)	7.43 (1.74) 68%	6.71 (2.02) 61%	0.086
Energy density score (max 15)	9.33 (1.92) 62%	9.6 (2.44) 64%	0.586
Stress score (max 15)	8.12 (1.65) 55%	8.19 (1.12) 55%	0.422
Knowledge score (m) M (SD)			
Total score (max 45)	27.86 (2.99) 62%	26.48 (6.47) 59%	0.38
Food pyramid score (max 11)	7.1 (1.64) 65%	6.19 (1.94) 56%	0.11
Energy density score (max 15)	9.14 (1.46) 63%	9.24 (2.49) 66%	0.88
Stress score (max 15)	8.05 (0.80) 55%	7.86 (1.90) 55%	0.675
Knowledge score (f) M (SD)			
Total score (max 45)	29.29 (4.08) 65%	29.38 (4.77) 65%	0.945
Food pyramid score (max 11)	7.76 (1.81) 71%	7.24 (2) 66%	0.379
Energy density score (max 15)	9.52 (2.32) 63%	9.95 (2.4) 66%	0.559
Stress score (max 15)	8.19 (1.17) 55%	8.52 (0.98) 55%	0.322

* p < 0.05; HNI: healthy nutrition index.

**Table 2:** Outcomes from intention to treat analysis regarding parents.

	FI T0 (*n* = 21)	FI T1 (*n* = 21)	FI T2 (*n* = 21)	CI T0 (*n* = 21)	CI T1 (*n* = 21)	CI T2 (*n* = 21)	FI–CI	Time	Group × Time
	M (SD)	M (SD)	M (SD)	M (SD)	M (SD)	M (SD)	Mean (SD)	F(2,40)	F(2,40)
	%	%	%	%	%	%	*p*	*p*	*p*
							(95% CI)	Eta²	Eta²
**Knowledge (both)**									
Total Score	27.83 (2.96)	29.77 (5.86)	30.07 (2.53)	27.11 (5.61)	30.98 (2.46)	29.91 (1.75)	0.11 (3.69)	**13.73**	1.37
(max 45)	62%	66%	67%	60%	69%	66%	*p = 0.846* [−1.03; 1.25]	p < 0.001 ** **0.156**	*p = 0.258* 0.018
Food pyramid	7.43 (1.81)	8.53 (1.40)	8.77 (1.00)	6.82 (1.97)	8.59 (1.02)	8.59 (1.05)	−0.24 (1.42)	**26.20**	0.99
(max 11)	68%	78%	80%	62%	78%	78%	*p = 0.265* [−0.68; 0.19]	p < 0.001 ** **0.261**	*p = 0.374* 0.013
Energy density	0.82 (0.14)	0.86 (0.20)	0.84 (0.16)	0.82 (0.21)	0.90 (0.11)	0.81 (0.14)	0.00 (1.62)	**3.35**	0.86
score (max 15)	5%	6%	6%	5%	6%	5%	*p = 0.895* [−0.05; 0.05]	p = 0.038 * **0.043**	*p = 0.426* 0.011
Stress score	8.07 (0.98)	8.43 (2.18)	7.96 (0.58)	8.28 (1.40)	8.46 (0.55)	8.30 (0.59)	0.19 (1.17)	1.76	0.39
(max 15)	54%	56%	53%	55%	56%	55%	*p = 0.289* [−0.17; 0.05]	*p = 0.176* 0.024	*p = 0.680* 0.005

* *p* < 0.05 ** *p* < 0.01.

**Table 3:** Time differences for parents.

Knowledge Scores	T0 vs. T1	T0 vs. T2	T1 vs. T2
	Mean (SD), *p* (95% CI)	Mean (SD), *p* (95% CI)	Mean (SD), *p* (95% CI)
**(Both)**			
Total	−2.90 (0.72), p < 0.001 ** [−4.66; −1.14]	−2.52 (0.56), p < 0.001 ** [−3.90; −1.14]	0.38 (0.50), *p = 1* [−0.85; 1.62]
Food pyramid	−1.43 (0.27), p < 0.001 ** [−2.08; −0.78]	−1.55 (0.24), p < 0.001 ** [−2.14; −0.95]	−0.12 (0.20), *p = 1* [−0.61; 0.37]
Energy density	−0.06 (0.03), *p = 0.115* [−0.12; 0.01]	−0.01 (0.03), *p = 1* [−0.07; 0.06]	0.05 (0.20), *p = 0.036* [0.00; 0.10]
Stress	−0.27 (0.20), *p = −612* [−0.77; 0.24]	0.04 (0.15), *p = 1* [−0.33; 0.42]	0.31 (0.17), *p = 0.228* [−0.11; 0.72]

** *p* < 0.01.

**Table 4:** Outcomes from intention to treat analysis regarding children.

	FI	FI	FI	FI	CI	CI	CI	CI	FI–CI	Time	Group × Time
	(*n* = 21) T0	(*n* = 21) T1	(*n* = 21) T2	(*n* = 21) T3	(*n* = 19) T0	(*n* = 19) T1	(*n* = 19) T2	(*n* = 19) T3	Mean (SD) *p*	F(2,40) *p*	F(2,40) *p*
									(95% CI)	Eta^2^	Eta^2^
**Knowledge scores**											
Total	27.24 (5.12)	38.00 (6.10)	37.53 (3.83)	37.35 (5.70)	27.67 (6.69)	37.00 (5.13)	35.48 (5.04)	34.71 (6.60)	−1.32 (8.98)	**47.52**	1.00
(max 60)	45%	63%	63%	62%	46%	62%	59%	58%	*p = 0.359* [−4.19; 1.56]	p < 0.001 ** 0.569	*p = 0.398* 0.027
Food pyramid	0.49 (0.17)	0.75 (0.20)	0.77 (0.15)	0.77 (0.15)	0.50 (0.11)	0.79 (0.16)	0.73 (0.15)	0.73 (0.13)	−0.10 (0.19)	**36.45**	0.92
(max 26)	2%	3%	3%	3%	2%	3%	3%	3%	*p = 0.745* [−0.07; 0.05]	p < 0.001 ** 0.49	*p = 0.432* 0.024
Energy density	0.55 (0.24)	0.74 (0.26)	0.77 (0.20)	0.75 (0.22)	0.59 (0.28)	0.66 (0.21)	0.70 (0.24)	0.64 (0.27)	−0.07 (0.32)	**9.40**	0.10
(max 15)	4%	5%	5%	5%	4%	4%	5%	4%	*p = 0.145* [−0.19; 0.03]	p < 0.001 ** 0.23	*p = 0.960* 0.003
Stress	0.64 (0.16)	0.66 (0.8)	0.64 (0.09)	0.67 (0.14)	0.71 (0.21)	0.70 (0.12)	0.63 (0.15)	0.65 (0.13)	0.02 (0.25)	2.09	2.28
(max 15)	4%	4%	4%	4%	5%	5%	4%	4%	*p = 0.955* [−0.07; 0.07]	*p = 0.106* 0.055	*p = 0.083* 0.6
**HNI** (RbP)	2.22 (0.44)	----	2.33 (0.50)	2.33 (0.50)	2.20 (0.45)	---	2.20 (0.45)	2.00 (0.00)	−0.16 (1.08) *p = 0.358*	0.20 *p = 0.817*	0.51 *p = 0.659*
									[−0.53; 0.21]	0.017	0.04
Score	9.11 (1.54)	----	9.56 (1.88)	9.67 (1.94)	9.20 (1.30)	---	9.40 (1.14)	8.60 (1.14)	−0.38 (4.43) *p = 0.602*	0.31 *p = 0.735*	0.78 *p = 0.470*
									[−1.92; 1.16]	0.025	0.061
Unfavorable %	0%		0%	0%	0%		0%	0%			
Neutral %	79%		66%	38%	59%		57%	87%			
Favorable %	21%		33%	62%	41%		43%	13%			
HNI (RbC)	2.43 (0.53)	----	2.29 (0.76)	2.14 (0.38)	2.80 (0.45)	---	2.80 (0.45)	2.80 (0.45)	**0.51 (1.26)** p = 0.025 *	0.24 *p = 0.792*	0.24 *p = 0.792*
									**[0.08; 0.95]**	0.023	0.023
Score	9.71 (2.06)	---	9.29 (3.68)	8.71 (1.38)	11.00 (1.22)	---	10.80 (2.77)	11.20 (1.92)	1.76 (6.32) *p = 0.109*	0.13 *p = 0.882*	0.29 *p = 0.752*
									[−0.47; 3.99]	0.012	0.028
Unfavorable %	36%		4%	0%	7%		0%	9%			
Neutral %	13%		41%	80%	43%		43%	45%			
Favorable %	50%		53%	20%	49%		57%	45%			
**Physical activity level**											
(RbP)	2.17 (0.75)	---	2.00 (0.00)	2.33 (0.52)	2.20 (0.84)	---	2.40 (0.89)	2.60 (0.55)	0.23 (1.71) *p = 0.413*	0.83 *p = 0.449*	0.29 *p = 0.755*
Score	0.33 (2.42)	---	0.67 (0.82)	1.67 (2.07)	0.60 (2.61)	---	1.00 (2.83)	3.00 (2.83)	0.64 (6.58) *p = 0.551*	2.92 *p = 0.080*	0.27 *p = 0.769*
									[−1.71; 3.00]	0.245	0.029
Low activity %	13%		10%	4%	17%		14%	23%			
Medium activity %	65%		41%	59%	59%		53%	33%			
High activity %	13%		50%	36%	24%		24%	44%			
(RbC)	1.42 (0.67)	---	2.00 (0.74)	1.83 (0.83)	1.93 (0.83)	---	2.00 (0.88)	2.14 (0.77)	0.27 (1.71) *p = 0.319*	3.72 *p = 0.032*	1.79 *p = 0.178*
									[−0.28; 0.83]	0.134	0.07
Score	−1.73 (2.82)	---	0.09 (2.59)	−0.09 (2.88)	−0.08 (2.78)	---	0.69 (3.54)	1.30 (3.82)	1.22 (7.02) *p = 0.288*	4.60 *p = 0.015*	0.52 *p = 0.600*
									[−1.10; 3.53]	0.173	0.023
Low activity %	52%		30%	47%	52%		47%	20%			
Medium activity %	26%		45%	22%	29%		26%	45%			
High activity %	13%		25%	30%	19%		26%	35%			
**Media consumption**											
Video/TV	1.42 (0.52)	---	1.33 (0.65)	1.50 (0.52)	1.20 (0.63)	---	1.20 (0.63)	1.10 (0.57)	−0.25 (1.33) *p = 0.255*	0.07 *p = 0.929*	0.50 *p = 0.501*
									[−0.70; 0.20]	0.004	0.034
≤0.5 h	100%		89%	95%	88%		100%	100%			
1–2 h	0%		11%	5%	12%		0%	0%			
≥3 h	0%		0%	0%	0%		0%	0%			
Computer	0.64 (0.63)	---	1.07 (1.07)	0.93 (0.73)	0.83 (0.72)	---	0.58 (0.67)	0.67 (0.65)	−0.19 (1.52) *p = 0.444*	0.16 *p = 0.849*	2.37 *p = 0.104*
									[−0.68; 0.31]	0.007	0.09
≤0.5 h	100%		93%	100%	100%		100%	100%			
1–2 h	0%		0%	0%	0%		0%	0%			
≥3 h	0%		7%	0%	0%		0%	0%			

RbP = Reported by Parents, RbC = Reported by Children, HNI = Healthy Nutrition Index; * *p* < 0.05 ** *p* < 0.01

**Table 5:** Time differences for children.

	T0 vs. T1	T0 vs. 2	T0 vs. T3	T1 vs. T2	T1 vs. T3	T2 vs. T3
	Mean (SD), *p*	Mean (SD), *p*	Mean (SD), *p*	Mean (SD), *p*	Mean (SD), *p*	Mean (SD), *p*
	(95% CI)	(95% CI)	(95% CI)	(95% CI)	(95% CI)	(95% CI)
Knowledge						
Total score	**−10.05 (1.31), ** *p* < 0.001 **	**−9.05 (0.97), ** p < 0.001 **	**−8.58 (1.21), ** p < 0.001 **	0.10 (0.73), *p = 1*	1.47 (0.83), *p = 0.507*	0.47 (0.75), *p = 1*
	**[−13.21; −6.89]**	**[−11.75; −6.35]**	**[11.97; −5.20]**	[−1.59; 3.05]	[−0.84; 3.77]	[−1.62; 2.56]
Food pyramid score	**−0.27 (0.40), ** *p* < 0.001 **	**−0.25 (0.03), ** p < 0.001 **	**−0.25 (0.03), ** p < 0.001 **	0.02 (0.03), *p = 1*	0.02 (0.03), *p = 1*	0.00 (0.02), *p = 1*
	**[−0.38; −0.16]**	**[−0.34; −0.16]**	**[−0.34; −0.16]**	[−0.05; 0.10]	[−0.05; 0.09]	[−0.06; 0.06]
Energy density score	**−0.18 (0.05), ** *p* = 0.007 *	**−0.22 (0.05), ** p < 0.001 **	−0.18 (0.06), p = 0.018 *	−0.03 (0.03), *p = 1*	0.01 (0.4), *p = 1*	0.04 (0.40), *p = 1*
	**[−0.33; 0.38]**	**[−0.35; −0.08]**	**[−0.33; −0.02]**	[−0.11; 0.05]	[−0.10; 0.12]	[−0.07; 0.15]
Stress score	**−0.01 (0.03), *p* = 1**	0.03 (0.03), *p = 1*	0.01 (0.04), *p = 1*	0.04 (0.02), *p = 0.86*	0.02 (0.02), *p = 1*	−0.03 (0.02), *p = 1*
	**[−0.1; 0.09]**	[−0.06; 0.13]	[−0.09; 0.12]	[−0.04; 0.09]	[−0.04; 0.08]	[−0.09; 0.04]
HNI RbP	---	−0.06 (0.18), *p = 1*	0.04 (0.16), *p = 1*	---	---	0.10 (0.14), *p = 1*
		[−0.55; 0.44]	[−0.385; 0.47]			[−0.27; 0.47]
HNI score	---	−0.32 (0.56), *p = 1*	0.02 (0.45), *p = 1*	---	---	0.34 (0.44), *p = 1*
		[−1.89; 1.25]	[−1.23; 1.28]			[−0.88; 1.56]
HNI RbC	---	0.07 (0.20), *p = 1*	0.14 (0.17), *p = 1*	---	---	0.07 (0.24), *p = 1*
		[−0.51; 0.66]	[−0.35; 0.64]			[−0.62; 0.77]
HNI score	---	0.31 (0.95), *p = 1*	0.40 (0.48), *p = 1*	---	---	0.09 (0.99), *p = 1*
		[−2.40; 3.03]	[−0.976; 1.78]			[−2.76; 2.93]
Physical activity RbP	---	−0.02 (0.24), *p = 1*	−0.28 (0.29), *p = 1*	---	---	−0.27 (0.20), *p = 0.678*
		[−0.72; 0.69]	[−1.12; 0.56]			[−0.87; 0.03]
Score	---	−0.37 (0.56), *p = 1*	−1.87 (0.99), *p = 0.277*	---	---	−1.50 (0.84), *p = 0.328*
		[−2.00; 1.27]	[−4.78; 1.04]			[−3.98; 0.98]
Physical activity RbC	---	−0.33 (0.14), *p = 0.079*	−0.31 (0.14), *p = 0.08*	---	---	0.01 (0.13), *p = 1*
		[−0.68; 0.03]	[−0.66; 0.03]			[−0.34; 0.36]
Score	---	−1.29 (0.51), *p = 0.056*	−1.15 (0.54), p = 0.032 *	---	---	−0.22 (0.56), *p = 1*
		[−2.61; 0.03]	**[−2.91; −0.11]**			[−1.68; 1.24]
Media Consumption						
Video/TV	---	0.04 (0.10), *p = 1*	0.01 (0.12), *p = 1*	---	---	−0.03 (0.12), *p = 1*
		[−0.24; 0.32]	[−0.29; 0.31]			[−0.35; 0.29]
Computer	---	−0.09 (0.17), *p = 1*	−0.06 (0.14), *p = 1*	---		0.03 (0.16), *p = 1*
		[−0.52; 0.35]	[−0.43; 0.31]			[−0.39; 0.45]

RbP = Reported by parents, RbC = Reported by children, HNI = Healthy Nutrition Index; * *p* < 0.05 ** *p* < 0.01

## Data Availability

All data are reported in the manuscript and the Supplementary Materials.

## Supplementary Materials

The following supporting information can be downloaded at: https://www.mdpi.com/article/10.3390/life12060779/s1; Supplementary File S1: Knowledge questionnaire.
